# Molecular Interaction of TPPP with PrP Antagonized the CytoPrP-Induced Disruption of Microtubule Structures and Cytotoxicity

**DOI:** 10.1371/journal.pone.0023079

**Published:** 2011-08-12

**Authors:** Rui-Min Zhou, Yuan-Yuan Jing, Yan Guo, Chen Gao, Bao-Yun Zhang, Cao Chen, Qi Shi, Chan Tian, Zhao-Yun Wang, Han-Shi Gong, Jun Han, Bian-Li Xu, Xiao-Ping Dong

**Affiliations:** 1 State Key Laboratory for Infectious Disease Prevention and Control, National Institute for Viral Disease Control and Prevention, Chinese Center for Disease Control and Prevention, Beijing, People's Republic of China; 2 Henan Provincial Center for Disease Control and Prevention, Zhengzhou, Henan Province, People's Republic of China; The Scripps Research Institute Scripps Florida, United States of America

## Abstract

**Background:**

Tubulin polymerization promoting protein/p25 (TPPP/p25), known as a microtubule-associated protein (MAP), is a brain-specific unstructured protein with a physiological function of stabilizing cellular microtubular ultrastructures. Whether TPPP involves in the normal functions of PrP or the pathogenesis of prion disease remains unknown. Here, we proposed the data that TPPP formed molecular complex with PrP. We also investigated its influence on the aggregation of PrP and fibrillization of PrP106–126 *in vitro*, its antagonization against the disruption of microtubule structures and cytotoxicity of cytosolic PrP in cells, and its alternation in the brains of scrapie-infected experimental hamsters.

**Methodology/Principal Findings:**

Using pull-down and immunoprecipitation assays, distinct molecular interaction between TPPP and PrP were identified and the segment of TPPP spanning residues 100–219 and the segment of PrP spanning residues 106–126 were mapped as the regions responsible for protein interaction. Sedimentation experiments found that TPPP increased the aggregation of full-length recombinant PrP (PrP23–231) *in vitro*. Transmission electron microscopy and Thioflavin T (ThT) assays showed that TPPP enhanced fibril formation of synthetic peptide PrP106–126 *in vitro*. Expression of TPPP in the cultured cells did not obviously change the microtubule networks observed by a tubulin-specific immunofluorescent assay and cell growth features measured by CCK8 tests, but significantly antagonized the disruption of microtubule structures and rescued the cytotoxicity caused by the accumulation of cytosolic PrP (CytoPrP). Furthermore, Western blots identified that the levels of the endogenous TPPP in the brains of scrapie-infected experimental hamsters were significantly reduced.

**Conclusion/Significance:**

Those data highlight TPPP may work as a protective factor for cells against the damage effects of the accumulation of abnormal forms of PrPs, besides its function as an agent for dynamic stabilization of microtubular ultrastructures.

## Introduction

Prion diseases, known as transmissible spongiform encephalopathies (TSEs), are fatal progressive neurodegenerative diseases characterized with spongiosis and neuronal loss in the central nervous system (CNS) [1], [2]. PrP is a glycosylphosphatidylinositol (GPI)-anchored membrane protein expressed mainly in CNS, whose normal function is still enigmatic. Those diseases are caused by the conversion of a host-derived cellular prion protein (PrP^C^) to the infectious scrapie prion protein (PrP^Sc^), a misfolded and proteinase K (PK)-resistant isoform, which represents the major component of infectious agent in brains [3], [4]. Although it is clear that PrP^Sc^ accumulates in the brain during most prion diseases, there is uncertainty about the mechanisms responsible for neuronal death.

Usually, PrP^C^ is anchored on the cell surface via a GPI moiety and begins its journey to the cell surface in endoplasmic reticulum (ER) [5], [6]. However, some PrP molecules are not co-translationally inserted into the ER and end up in the cytosol [7]. Cytosolic PrP accounts for a minor intracellular subset of PrP^C^ that has attracted much attention because its accumulation sensitizes cell to death [8]. Recent study proposes the evidence that cytosolic PrP is neurotoxic and may play a role in the neurodegeneration of prion disease. [9]. Cytosolic PrP, either from retro-translocation [10] or from the impaired import into the ER [11], is destined for proteasome degradation. Moreover, changes of the normal secreting and maturing pathway of wild-type (WT) PrP will cause obvious cytotoxic activity in the cultured cells, possibly due to the generation of intracellular PrP. The accumulation of PrP in the cytosol (CytoPrP) may trigger cell apoptosis by mitochondrial relative apoptosis pathway [12].

PrP shows characteristics to interact with microtubular cytoskeleton and its major component, tubulin [13]–[15]. Microtubules are cellular structures that play a central role in intracellular transport, metabolism, and cell division. Interference with microtubule dynamics leads to mitotic arrest and initiation of apoptosis [16]. Our previous studies have also confirmed that the expressed cytosolic PrP is able to interact with endogenous tubulin, and that accumulation of cytosolic PrP apparently disrupts the microtubule network in the cultured cells and reduces the cell viability via inducing apoptosis [17].

TPPP/p25 (tubulin polymerization promoting protein/p25) is a recently discovered, brain-specific unstructured protein involved in brain function [18]–[20]. It is found predominantly in oligodendrocytes in normal brain but is enriched in neuronal and glial inclusions of Parkinson's disease and other synucleinopathies [21], [22]. Its physiological function seems to be the dynamic stabilization of microtubular ultrastructures, as well as the projections of mature oligodendrocytes and ciliary structures [23]–[25]. Microtubules, which form a major part of the cytoskeleton, display many physiological functions in eukaryotic cells. The dynamic reorganizing ability and stability of microtubular systems show great variability in different tissues and at different stages of tissue development. The distinct functions of microtubular structures are attained through static as well as variable association with different proteins. Therefore, TPPP is a new microtubule-associated protein (MAP). Lehotzky and his colleagues have reported that dynamic targeting of microtubules by TPPP affects cell survival [26]. A number of proteins have been identified as potential binding partners of TPPP under physiological or pathological conditions [27]–[32]. However, whether TPPP is able to form complex with PrP and what its contributions to physiological functions of PrP^C^ and pathogenesis of prion disease are remain unsettled.

In the present study, we proposed the molecular evidences of TPPP interacting with PrP, and firstly characterized the segments of TPPP spanning residues 100–219 and the segments of PrP spanning residues 106–126 as their interacting regions. Under the experimental condition, recombinant TPPP enhanced the aggregation of the full-length PrP and efficiently induced fibril formation of peptide PrP106–126. Expression of TPPP in cultured cells did not influence the microtubule network and cell viability apparently, but antagonized the disruptive effect of cytosolic PrP on microtubule structures and enhanced the cellular resistant activities to the cytotoxicity of CytoPrP. Moreover, the levels of the endogenous TPPP in the brains of scrapie-infected experimental hamsters were significantly decreased. It highlights an essential role of TPPP in maintaining the dynamic stabilization of microtubular ultrastructures, meanwhile, as well as a possible intermediate character in the pathogenesis of TSEs.

## Results

**Figure 1:**
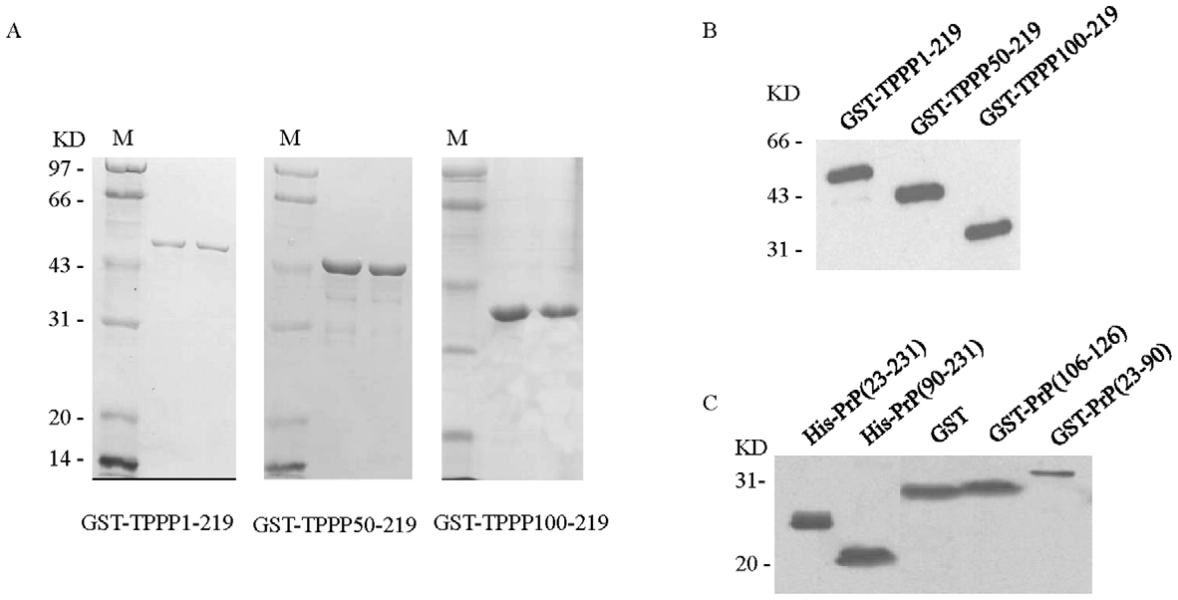
Protein purification of various recombinant TPPP and PrP proteins. A. 15% SDS-PAGE assay for various purified recombinant TPPP proteins. M: standard molecular weights of protein. B. Western blot assay for various recombinant TPPP proteins with anti-TPPP mAb. C. Western blot assay for various recombinant PrP proteins with different antibodies. His-PrP(23–231) and His-PrP(90–231) were blotted with anti-PrP pAb. GST, GST-PrP(106–126) and GST-PrP(23–90) were blotted with anti-GST mAb. Protein molecular markers are shown on the left.

With a RT-PCR technique, a 657 bp cDNA fragment corresponding to the full-length human TPPP (aa 1–219) was amplified, and subsequently two shorter DNA sequences encoding the truncated TPPP (TPPP50–219 and TPPP100–219) were generated. Various recombinant TPPP proteins were expressed and purified from the transformed *E. coli*. SDS-PAGE showed that the purified recombinant GST-TPPP proteins were at the positions as expected, respectively (Fig. 1A). Western blots with anti-TPPP mAb demonstrated clear reactive bands at the expected positions (Fig. 1B). Various recombinant PrP proteins were expressed and purified and Western blots with anti-PrP pAb or anti-GST mAb demonstrated the bands at the expected positions clearly (Fig. 1C).

### Molecular interaction between TPPP and PrP

**Figure 2:**
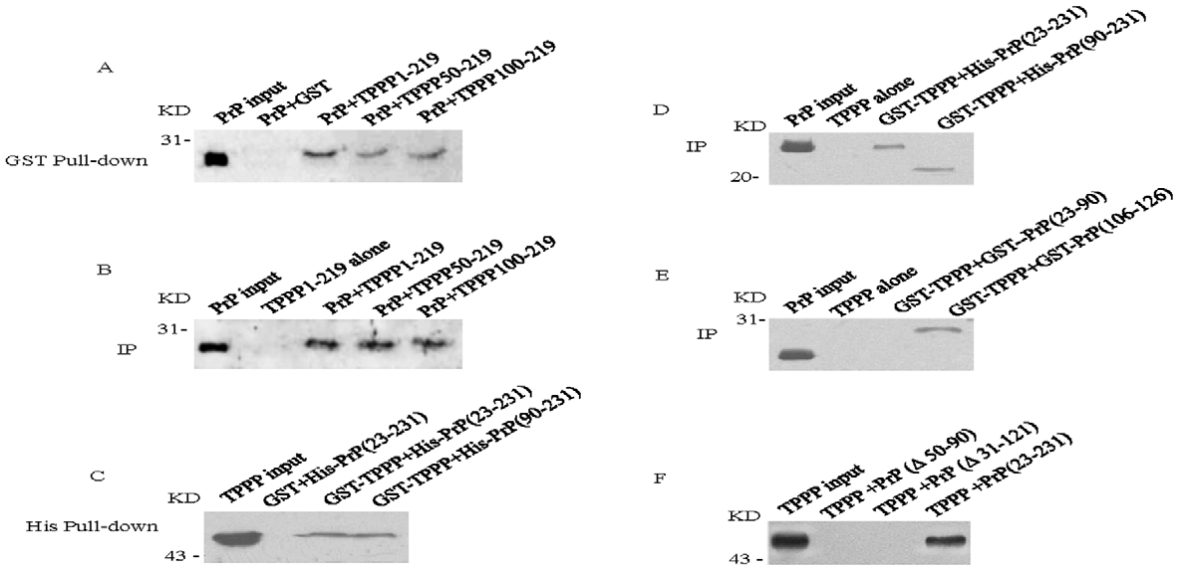
Molecular interactions between various recombinant TPPP and PrP. A. GST pull-down assay of various recombinant TPPP with His-PrP23–231. The TPPP-PrP complexes were precipitated with glutathione agarose beads. The bound PrP was detected by PrP specific 3F4 mAb. B. Co-immunoprecipitation assay of various TPPPs with His-PrP23–231. The TPPP-PrP complexes were precipitated by anti-TPPP pAb and the bound PrP was detected with PrP specific 3F4 mAb. C. His pull-down assay of various His-tagged PrPs with GST-TPPP1–219. The TPPP-PrP complexes were precipitated with Ni-NTA agarose beads. The bound TPPP was detected by anti-TPPP pAb. D. Co-immunoprecipitation assay of various His-tagged PrPs with GST-TPPP1–219. The TPPP-PrP complexes were precipitated by anti-TPPP pAb and the bound PrP was detected with PrP specific 3F4 mAb. E. Co-immunoprecipitation assay of various GST-tagged PrP with GST-TPPP1–219. The TPPP-PrP complexes were precipitated by anti-TPPP mAb and the bound PrP was detected with anti-PrP pAb. F. Co-immunoprecipitation assay of various PrPs in the context of full-length with TPPP1–219. The TPPP-PrP complexes were captured by anti-PrP pAb and the bound TPPP was detected with anti-TPPP mAb. PrP-input represents the purified PrP23–231 and TPPP-input represents the purified GST-TPPP1–219, directly loaded as controls of Western blots. Protein molecular markers are shown on the left.

Previous studies have suggested a number of proteins as potential binding partners of TPPP [27]. In order to address the possible interaction between TPPP and PrP, and then, to determine the possible binding regions for interaction within TPPP and PrP, various recombinant TPPPs, including GST-TPPP1–219, GST-TPPP50–219 and GST-TPPP100–219, was mixed separately with PrP23–231 in the GST pull-down assays, while the recombinant GST protein was used as control. Western blot of the eluted products with anti-PrP mAb 3F4 demonstrated clear reactive bands in all the reactions containing GST-TPPP proteins, but not in that of control with GST (Fig. 2A). Co-immunoprecipitations of the mixtures of TPPP and PrP23–231 using anti-TPPP pAb as the capturing antibody also revealed PrP-specific signals in the Western blots using mAb 3F4 as the detecting antibody in all the preparations (Fig. 2B). It implies that TPPP can form complex with PrP *in vitro* and the region(s) within TPPP responsible for the interaction with PrP locate likely at C-terminus of TPPP spanning residues 100–219.

To map the possible position within PrP interacting with TPPP, a series of truncated PrPs, including GST-PrP23–90, GST-PrP106–126 and His-PrP90–231, were incubated with the full-length TPPP, respectively. His pull-down assays identified clear TPPP protein complexes in the reactions of His-PrP23–231 and His-PrP90–231, but not in that of the control with His-GST (Fig. 2C). Co-immunoprecipitation assay showed TPPP-PrP protein complexes in the preparations of His-PrP23–231 and His-PrP90–231, which were precipitated with anti-TPPP pAb and detected with mAb 3F4 (Fig. 2D). Co-immunoprecipitation of GST-PrP with anti-TPPP mAb also revealed the PrP protein complexes in the preparations of GST-PrP106–126, whereas not in that of GST-PrP23–90, after blotted with PrP-specific pAb (Fig. 2E). Furthermore, two GST-PrP mutants deleted the segments of aa50–90 (Δ50–90) and aa31–121 (Δ31–121) in the context of full-length PrP (aa23–231), as well as GST-PrP23–231 were subjected into immunoprecipitation assays with the full-length TPPP, using anti-PrP pAb as the capturing antibody and anti-TPPP mAb as the detecting antibody. Clear protein complex was observed in the reaction of PrP23–231, but not in that of PrPΔ50–90 or PrPΔ31–121 (Fig. 2F). It suggests that the region within PrP responsible for the interaction with TPPP may locate at the segment spanning residues 106–126.

**Figure 3:**
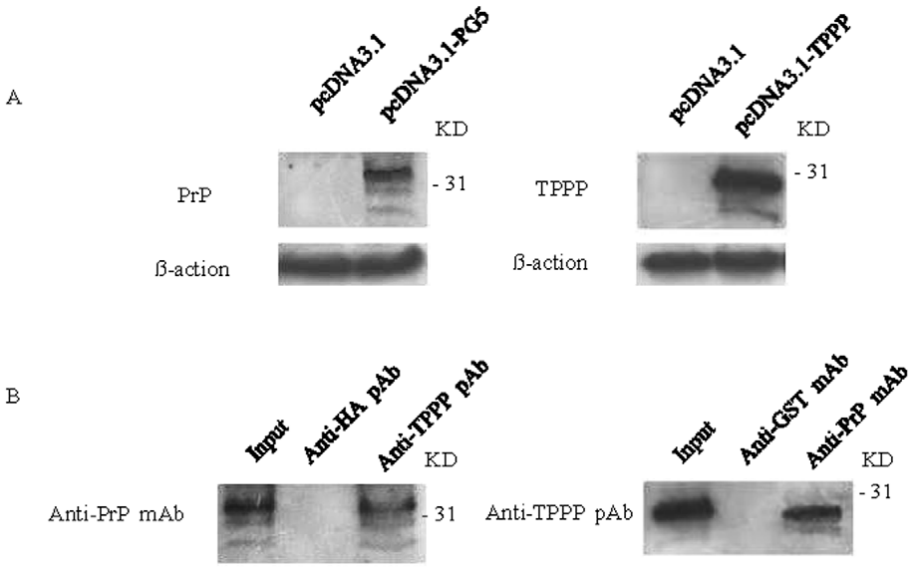
Molecular interactions between the expressed PrP and TPPP in HeLa cells. A. Western blot analyses of the expressions of PrP (left panel) and TPPP (right panel) in the HeLa cells transfected with or without PrP or TPPP expressing plasmid. Cells were harvested 48 h post-transfection and cell lysates were separated onto 12% SDS-PAGE. B. Co-immunoprecipitation assays of the expressed PrP and TPPP in HeLa cells. The PrP-TPPP complexes in cell lysates were captured by anti-TPPP pAb and blotted by anti-PrP mAb (left panel), or captured by anti-PrP mAb and blotted by anti-TPPP pAb (right panel). Protein molecular markers are shown on the right.

To see the potential PrP-TPPP interaction in cells, cell line HeLa, which was confirmed to have undetectable endogenous PrP and TPPP in Western blots (Fig. 3A), were transfected with the plasmids expressing the full-length wild-type PrP (pcDNA3.1-PG5) and the full-length TPPP (pcDNA3.1-TPPP). Distinct PrP or TPPP signals were detected in the cell lysates after blotted with respective antibodies (Fig. 3A). After co-transfected with pcDNA3.1-PG5 and pcDNA3.1-TPPP, the cell lysates were employed into immunoprecipitation assays, either captured with anti-TPPP pAb and detected with anti-PrP mAb (Fig. 3B, left panel), or captured with anti-PrP pAb and detected with anti-TPPP pAb (right panel). In both assays, PrP-TPPP complexes were identified in the preparations precipitated with specific antibodies, but not in that with isotype antibodies (Fig. 3B). It indicates that the *de novo* expressed PrP and TPPP form molecular complex in the cells.

### TPPP enhanced the precipitation of rPrP and fibril formation of PrP106–126 *in vitro*

**Figure 4:**
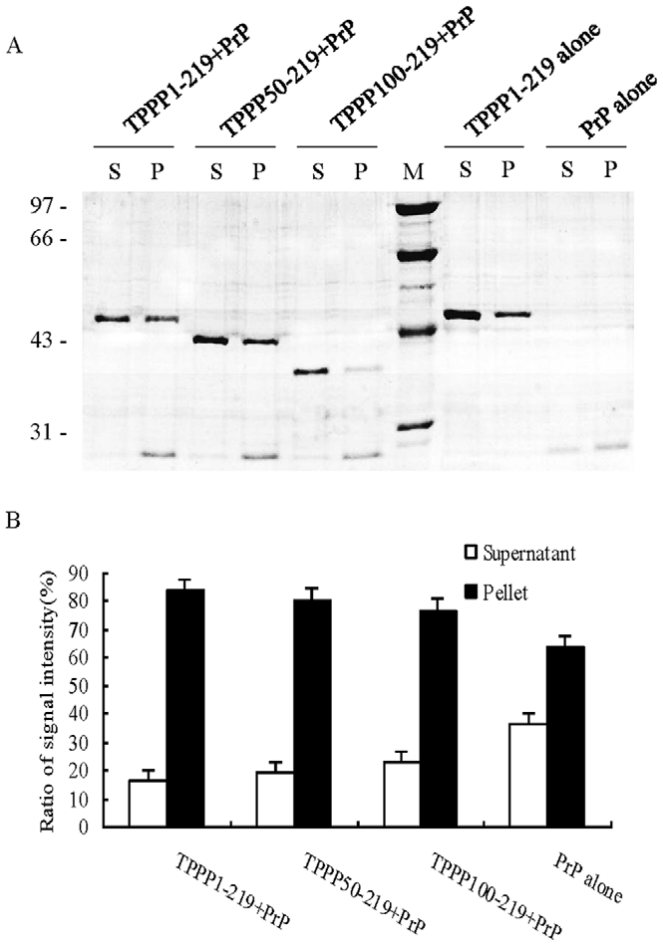
Evaluation of the sedimentation of PrP in the presences of various recombinant TPPPs. A. Sedimentation of PrP in the condition of favoring fibril formation. 0.5 mg/ml PrP was incubated with GST-TPPP1–219, GST-TPPP50–219 and GST-TPPP100–219, respectively, at an 1∶2 (PrP∶TPPP) molar ratio. TPPP alone and PrP alone were processed with the same protocol in the polymerization buffer as controls. The preparations were separated in a 15% SDS-PAGE and stained with Coomassie brilliant blue. S and P represent as supernatant and pellet after centrifuging, respectively. B. Comparative analyses of sedimentation of PrP in the presences of various recombinant TPPP by densitometry. PrP signals on Figure 3A were quantified with computer-assisted software Image TotalTech. The total signal intensities of PrP in supernatant and pellet were summated and defined as 100%. The percentages of each sample were calculated and given as ratios of the signal intensity. The average values were calculated from three independent tests and presented as mean ± SD.

To investigate the potential influence on the stability of PrP after interacting with TPPP *in vitro*, PrP23–231 solutions were incubated with three different TPPP proteins at 37°C for 1 h, while PrP23–231 as well as TPPP1–219 incubated alone was applied as controls. SDS-PAGE revealed that predominate portions of PrPs incubated with three TPPP constructs existed in the pellet fractions (Fig. 4A). Comparative analyses of the ratios of the PrP signal intensities in the fractions of supernatant and pellet each preparation demonstrated apparent increases of the signal intensities in fractions of pellets treated with TPPPs, showing statistical difference compared with that of PrP alone (P<0.05, Fig. 4B). It seems that TPPP facilitates the aggregation of PrP *in vitro*.

**Figure 5:**
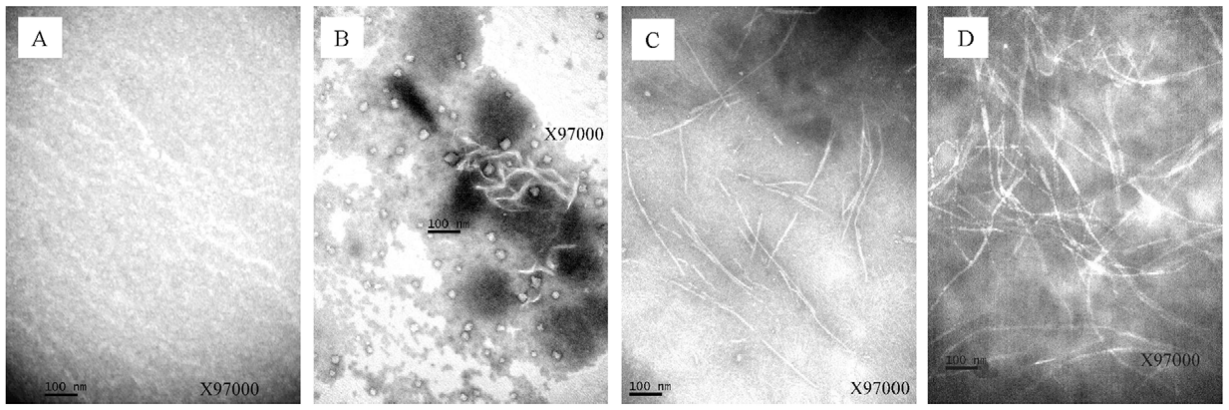
Morphological assays of the effects of various recombinant TPPP proteins on the fibril formation of synthetic PrP106–126 *in vitro* with a transmission electronic microscopy. 0.5 mg/ml synthetic peptide PrP106–126 was incubated in the absence (A) or presence of TPPP1–219 (B), TPPP50–219 (C) and TPPP100–219 (D) at 37°C for 72 h, respectively. The molar ratio of PrP106–126 to TPPP was 2∶1. Magnification was 97,000×. Scale bar represented 100 nm.

PrP spanning residues 106–126 seems to be responsible for the molecular interaction between PrP and TPPP. In order to evaluate the influences of TPPP on the fibrillization of PrP106–126 morphologically, the synthetic peptide PrP106–126 incubated with or without TPPP were applied on copper grids for phosphotungstinic acid negative staining and subjected to transmission electron microscopy. Fibrils were easily observed in the preparations of synthetic PrP106–126 treated with GST-TPPP1–219, GST-TPPP50–219 and GST-TPPP100–219 (Fig. 5B to D), whereas almost no fibrils were detectable in the micrographs of respective synthetic PrP106–126 without TPPPs (5A). Additionally, more and larger fibril masses seemed to be frequently seen in the preparations of GST-TPPP50–219 and GST-TPPP100–219 than that of GST-TPPP1–219 (Fig. 5). It indicates that TPPPs can enhance the fibril formation of peptide PrP106–126 *in vitro*, in which its C-terminus spanning residues 100–219 may play a more important role.

**Figure 6:**
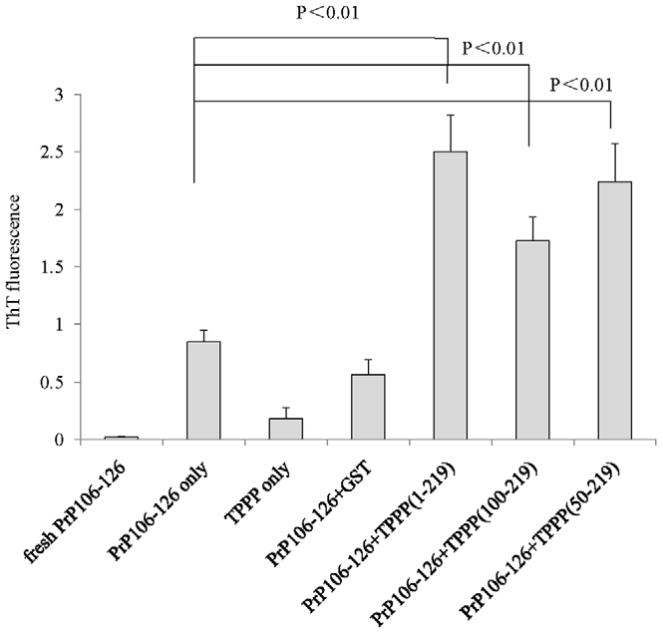
The ThT assays of the effect of TPPP on the fibril formation of synthetic PrP106–126 *in vitro*. 0.5 mg/ml synthetic peptide PrP106–126 was incubated alone, or with GST or with various GST-TPPP proteins at 37°C for 72 h, respectively. The molar ratio of PrP106–126 to TPPP was 2∶1. The average values are calculated from three independent tests and presented as mean ± SD.

To get more evidences of the enhancement of TPPP on the fibrillization of PrP106–126, freshly dissolved PrP106–126 were mixed with various recombinant TPPP proteins at RT for 72 h, while PrP106–126 alone, TPPP1–219 alone and PrP106–126 mixed with GST were employed in the same experimental process as controls. In line with our previous results [38], incubation of PrP106–126 alone led to an increase of ThT fluorescent value (mean value was 0.847) compared with the freshly dissolved PrP106–126 (mean value was 0.02, Fig. 6). Incubation of PrP106–126 with the full-length TPPP (1–219) and with two N-terminal truncated constructs (TPPP50–219 and TPPP100–219) induced 2 to 3-fold increased ThT fluorescent values, showing significantly statistic difference (P<0.01), whereas incubation with GST did not alter the OD value obviously (Fig. 6). It demonstrates again the enhancement of TPPP on the fibrillization of PrP106–126 *in vitro*.

### TPPP antagonized the inhibitive effect of CytoPrP on cellular microtubule structures

**Figure 7:**
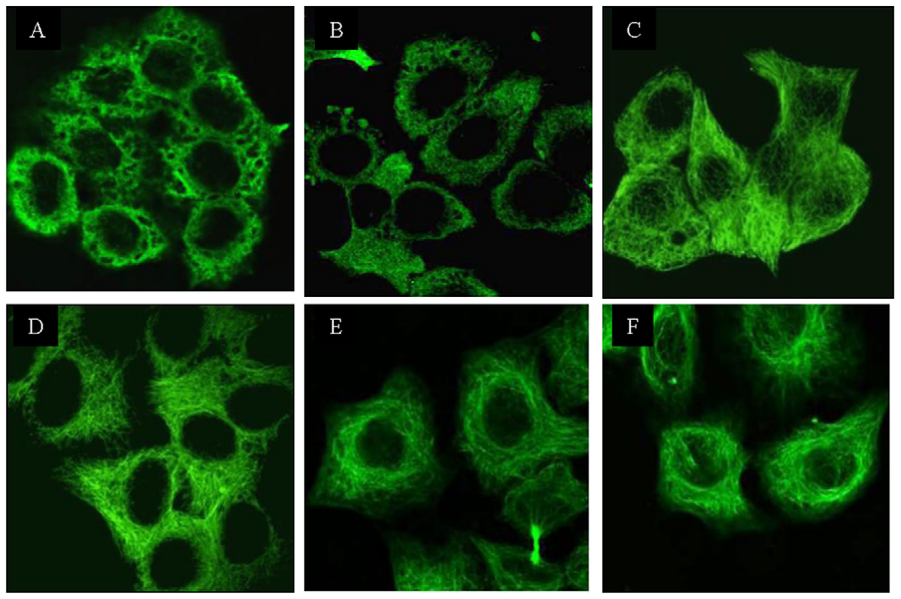
Morphological analyses of the influences of TPPP and PrP on the structures of microtubule in HeLa cells by a confocal microscopy. The structures of microtubules of the cells treated with various agents were monitored 48 h post-transfection. Panel A: colchicines (10 µM). Panel B: pcDNA3.1-CytoPrP. Panel C: pcDNA3.1-CytoPrP and pcDNA3.1-TPPP. Panel D: pcDNA3.1. Panel E: pcDNA3.1-PG5. Panel F: pcDNA3.1-TPPP.

Previous studies have reported that accumulation of cytosolic PrP acts as a microtubule-disrupting agent to influence the integrity of the microtubule network [17]. TPPP, tubulin polymerization promoting protein, is named by its functional characteristics of dynamic stabilization of microtubular ultrastructures [18], [19]. Different plasmids, including pcDNA3.1-PrP23–230 expressing CytoPrP, pcDNA3.1-PrP-PG5 expressing wild-type full-length PrP, pcDNA3.1-TPPP1–219 and blank vector pcDNA3.1 were transfected or co-transfected into HeLa cells and the cellular microtubule network were examined 48 h post-transfection by immunofluorescent staining with the tubulin specific mAb. Confocal microscopy assays revealed clear networks of microtubule in the HeLa cells receiving pcDNA3.1 (Fig. 7D) and severe disruptions of microtubule structures in the cells treated with colchicines, a microtubule-disrupting agent (7A). The microtubule structures in the cells transfected by pcDNA3.1-PrP-PG5 (Fig. 7E) or pcDNA3.1-TPPP1–219 (7F) remained almost intact. As expected, in the cells expressing cytosolic PrP almost no fibril structure was observable in cytoplasm instead of different sizes of vacuolation, (Fig. 7B), whereas the microtubule structures in that co-transfected with pcDNA3.1-TPPP1–219 were clearly observable (Fig. 7C). It suggests that TPPP may act as an agent to antagonize the inhibiting effect caused by the accumulation of cytosolic PrP on the formation of microtubule.

### TPPP antagonized the cytotoxicity of CytoPrP on the cultured cells

**Figure 8:**
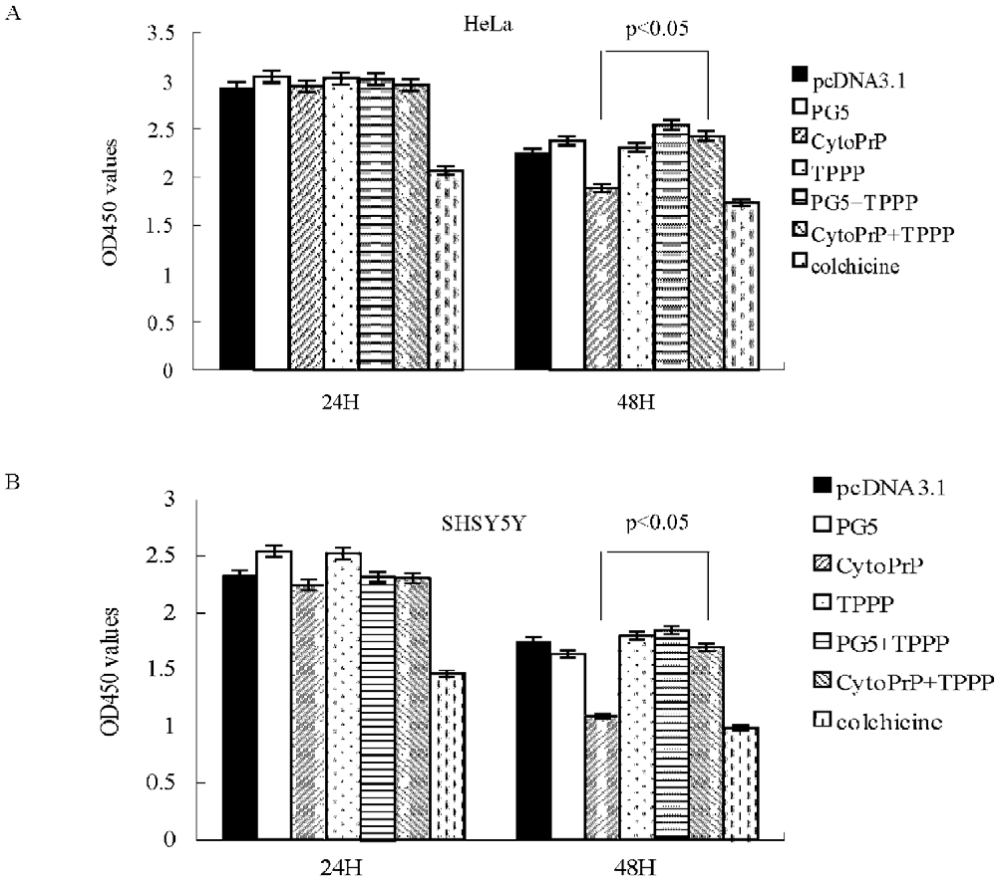
Analyses of cell viabilities receiving various plasmids expressing PrPs and/or TPPP. The cell viabilities of HeLa (A) and SHSY5Y (B) cells transfected with various plasmids were measured by a commercially Cell Counting Kit 24 and 48 h after transfection. Cells treated with colchicines (10 µM) were used as controls. The average data of each preparation was calculated based on three independent experiments and represented as mean ± S.D.

To assess the possible effects of expressing TPPP and PrP on cell viability, plasmids expressing TPPP, CytoPrP and PrP-PG5 were transiently transfected, alone or together, into HeLa and SHSY5Y cells. CCK-8 assays revealed almost same patterns in those two cell lines (Fig. 8). The cell viabilities in all preparations 24 h after transfection maintained quite comparable, except that treated with colchicines. In line with the disruption of microtubule network, remarkably low cell viabilities were observed in the cells expressing cytosolic PrP 48 h after transfection, whereas higher viabilities in the cells co-transfected with pcDNA3.1-CytoPrP and pcDNA3.1-TPPP, showing significant difference (P<0.05) (Fig. 8). Meanwhile, expressions of PrP-PG5 or TPPP alone did not change the cell growth ability significantly 48 h post-transfection. It demonstrates that accumulation of cytosolic PrP in cytoplasm causes marked cytotoxicity and expression of TPPP efficiently antagonizes the cytotoxicity of CytoPrP, possibly via maintaining the dynamic stabilization of microtubular ultrastructures.

### The levels of TPPP in the brains of scrapie experimental animals lowered dramatically at the terminal stages

**Figure 9:**
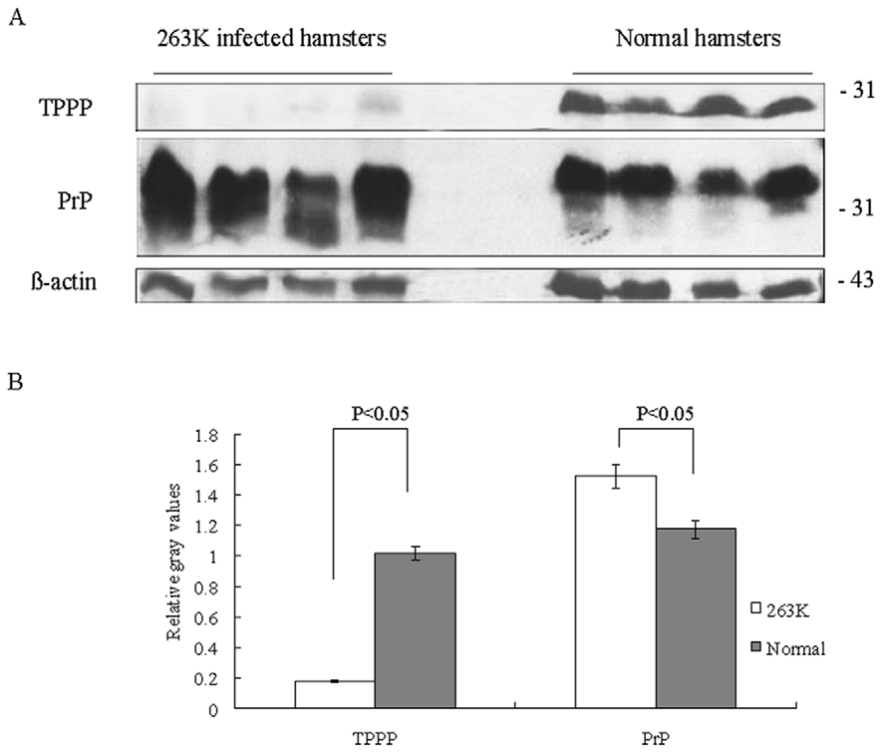
Comparative analyses of the levels of TPPP and PrP in the brain tissues of normal and scrapie agent 263K-infected hamsters collected at the moribund stage. A. Western blot assays for the endogenous TPPP and PrP with anti-TPPP pAb and mAb 3F4, respectively. Same amounts of individual brain homogenate were loaded in 12% SDS-PAGE. The scrapie infected as well as normal hamsters were indicated on the top of the graphs. The specific immunoblots of TPPP, PrP and β-actin were indicated on the left and molecular mass markers were indicated to the right. B. Quantitative analyses of each average grey value of the preparations after equilibrated with that of individual β-actin. The average grey values were calculated from four scrapie infected hamsters and four normal ones and presented as mean ± SD. Statistical differences compared with normal controls were illustrated as P<0.05.

To see the situation of TPPP in brain tissues of scrapie experimental animals, the brain homogenates of four scrapie agents 263K-infected hamsters, which were collected at the terminal stage of clinical course, were employed into TPPP and PrP specific Western blots. Four health hamsters of 80-day old were sampled as the control. Compared with that of the health control, the intensities of PrP specific blots were greatly high while that of TPPP-specific signals were extremely low in the preparations of scrapie infected animals (Fig. 9A). Quantitative analyses of the relative gray values of the immunoblot images after equilibrated with that of β-actin identified significantly increased PrP and decreased TPPP in the brains of scrapie infected hamsters, compared with that of normal one (P<0.05, Fig. 9B). It suggests that deposits of PrP^Sc^ in brains of scrapie experimental hamsters cause a marked decrease of endogenous TPPP at their final stage of clinical courses.

## Discussion

Microtubule networks are critical elements in a variety of fundamental functions, including intracellular scaffolding, cell division, secretory processes, regulatory of cellular motility and transport. Microtubules can be stabilized by structural microtubule-associated proteins (MAPs), such as tau that stimulates microtubule assembly [39], [40]. TPPP had been found as an impurity component of a tau kinase fraction [41]. The functional characteristics of TPPP resemble those of MAPs and denote it as tubulin polymerization promoting protein (TPPP) [18], [19]. Its physiological function seems to be the dynamic stabilization of microtubular ultrastructures, as well as the projections of mature oligodendrocytes and ciliary structures [23]–[25]. A number of proteins have been identified as potential binding partners of TPPP, some of these have been reported as targets under physiological or pathological conditions, such as proteins involved in signal transduction Cdk5, GSK-3, protein kinase A [32], structural protein tubulin [30], and the pre-synaptic protein a-synuclein [29], etc. In this study, we have firstly proved the molecular interaction between TPPP and PrP, and subsequently identified the segments of TPPP spanning residues 100–219 and the segments of PrP spanning residues 106–126 as the interacting regions for those two proteins.

Disfunction of microtubule is repeatedly described in some neurodegenerative diseases, including human and animal prion diseases [42], [43]. Our previous studies have confirmed that PrP is able to binding with cellular tubulin through its N-terminus and WT-PrP does not influence the formation of microtubule from tubulin *in vitro* and the normal microtubule structure in cultured cells [17], [44]. On the other hand, changes of PrP sequences, e.g. fCJD related PrP mutants with extra octarepeats insertions, and changes of subcellular position of PrP in cells, e.g. accumulation of cytosolic PrP in cytoplasm, result in not only strong inhibition on microtubule assembly *in vitro*, but also apparent disruption of microtubule structures in cells, which are largely related with the final phenomenon of cell apoptosis [17], [44]. Those abnormalities of cellular microtubule structures are believed to be related with the molecular interaction between PrP and tubulin, as the octarepeat-insertional PrPs and CytoPrP show apparently binding capacities with tubulin, even that the octarepeat-insertional PrPs possess stronger binding activities [17], [44]. Although expressions of TPPP and WT-PrP, separately or together, affects neither the microtubule structure nor cell viability, our data here have provided the evidences that presence of TPPP does antagonize the disruptive effect on cellular microtubule structure and the cytotoxicity of cytosolic PrP. It may suggest that TPPP, besides its function as an agent for dynamic stabilization of microtubular ultrastructures, works as a protective factor for cells against the damage effect of the accumulation of abnormal CytoPrP.

Through the molecular interaction, TPPP shows apparent enhancement on the aggregation of the full-length recombinant PrP *in vitro* and fibril formation of synthetical peptide PrP106–126. Those phenomena seem to be more remarkable in the preparations of C-terminal constructs of TPPP (TPPP50–219 and TPPP100–219), which contain the region(s) for interacting with PrP. It has been described that in addition to the full-length TPPP (TPPP/p25), there are two shorter paralogs, TPPP2/p18 and TPPP3/p20, in the human TPPP group, which lack of the unfolded N-terminal tail [45]. Study has reported that TPPP3/p20 displays more extensive microtubule-binding activity than TPPP/p25 [45]. Those data, taking together with our data in this study, highlight that the C-terminus of TPPP is more active region for interacting with other biologically functional agents. Segment PrP106–126 is an active domain of PrP, showing some PrP^Sc^-like characteristics and a notable toxicity on neurons [46]. Our other studies have identified that the cytotoxicity of PrP106–126 lies on its molecular composition, and that cytotoxicity appears in the form of monomer but disappears in the form of fibril [38]. Meanwhile, PrP106–126 is an active domain, which interacts with many metal ions [47] and nucleic acid [48], as well as Hsp104 and 14-3-3 in our studies (Han et al, in preparation). Therefore, it is likely to speculate that the protective activity of TPPP against the cytotoxicity of CytoPrP, even other abnormal forms of PrPs, might partially undergoes its enhancement for formation of non-toxigenic aggregated PrP from toxigenic monomer PrP.

A significantly decreased endogenous TPPP in the brains of scrapie experimental animals has been proposed in this study, which corresponds well the previous observation of lower levels of endogenous tubulin in the brain tissues of scrapie-infected hamsters [17]. Remarkable changes of microtubule associated protein tau, regardless of the level or phosphorylating pattern, as well as the relevant kinases, have been also described in the brains of a serial of naturally-occurred human and animal TSEs [49] and scrapie experimental animals [36]. Additionally, tau and tubulin have been already addressed as PrP-, even PrP^Sc^-, interacting partners [44], [49]. Those observations definitely point to that damage or destroy of microtubule structures in neuron is an intermediate or a final event during the pathogenesis of TSEs.

## Materials and Methods

### Ethics statement

Animal experiment in this study was approved by the Experimental animal Ethical Committee of National Institute for Viral Disease Prevention and Control, China CDC under protocol 2009ZX10004-101. All the Chinese golden hamsters were maintained under clean grade. Housing and experimental protocols were in accordance with the Chinese Regulations for the Administration of Affairs Concerning Experimental Animals.

### Plasmid construction

The single-stranded cDNA was purchased from the OriGene Technologies Company. With a PCR technique, the cDNA sequence encoding human TPPP was amplified with the forward primer (5′GCGGATCCATGGCTGACAAGGCC3′, with a BamHI site underlined) and the reverse primer (5′ GCGAATTCCTACTTGCCCCCTTGC3′, with a EcoRI site underlined). After verified by sequencing assays, the full-length human TPPP (aa 1–219) was cloned into a prokaryotic expressing vector pGEX-6p-GST, yielding pGEX-6p-GST-TPPP(FL). Two other sequences encoding truncated mutants of TPPP, including TPPP50–219 and TPPP100–219 were generated by PCR using the plasmid pGEX-6p-GST-TPPP(FL) as the templates. The PCR product was subsequently inserted into a prokaryotic expressing vector pGEX-6p-GST, generating recombinant plasmids pGEX-6p-GST-TPPP(50–219) and pGEX-6p-GST-TPPP(100–219). The plasmids expressing His-fusion PrPs, including pQE30-His-PrP for PrP from aa 23 to 231 and pQE30-His-PrP(90–231) for PrP from aa 90 to 231, and plasmids expressing GST-fusion PrPs, including pGEX-2T-GST-PrP(23–90) for PrP from aa 23 to 90 and pGEX-2T-GST-PrP(106–126) for PrP from aa 106 to 126, as well as three plasmids expressing the PrPs in the context of full-length segment (aa23–231), including pGEX-2T-GST-PrP(23–231) for PrP from aa 23 to 231, pGEX-2T-GST-PrP(Δ50–90) for PrP deleted from aa 50 to 90 and pGEX-2T-GST-PrP(Δ31–121) for PrP deleted from aa 31 to 121, were constructed previously [33], [34].

To construct the mammalian expressing plasmid encoding the full-length TPPP, TPPP sequence was inserted into vector pcDNA3.1, generating pcDNA3.1-TPPP(FL). The plasmid pcDNA3.1-PrP-PG5 expressing the full-length wild-type (WT) human PrP (aa 1–253) with five octarepeats (PG5) [35] and pcDNA3.1-CytoPrP expressing human PrP protein from amino acid aa 23 to 230 [12], were constructed previously.

### Protein expression and purification

The recombinant prokaryotic proteins tagged with GST or His were bacterially expressed in *E. coli* strains M15 or BL21. GST-fusion proteins were purified with Glutathione Sepharose 4B (Amersham Pharmacia) and His-tagged proteins were purified with Ni-NTA Agarose (Qiagen, Germany) according to the protocol described in our previous study [33]. The purity of each protein was verified by SDS-PAGE and immunoblot. Protein concentrations were determined by a BCA method (Qiagen, Germany).

Human PrP106–126 peptide (KTNMKHMAGAAAAGAVVGGLG) was synthesized by Invitrogen (USA).

### Western blots

The tested recombinant proteins and brain homogenates were separated by 15% SDS-PAGE and electro-transferred onto nitrocellulose membranes. After blocking with 5% nonfat-dried milk in PBS (phosphate buffered saline, pH 7.6) overnight at 4°C, the membranes were incubated with 1∶5,000 PrP specific monoclonal antibody (mAb) 3F4 (Deko, Denmark), 1∶1,000 anti-human β-actin mAb (Santa Cruz, USA), 1∶2,000 anti-TPPP mAb (Santa Cruz, USA) or 1∶1,000 anti-TPPP protein polyclonal antibody (pAb, Santa Cruz, USA) for 2 h at room temperature. After washing with PBST (phosphate buffered saline, pH 7.6, containing 0.05% Tween-20), the membranes were incubated with 1∶5,000 horseradish peroxidase (HRP)-conjugated anti-mouse or anti-rabbit. The reactive signals were visualized by ECL kit (PE Applied Biosystems, Foster City, USA).

### Pull-down assay

To identify the potential interaction between PrP and TPPP, 5 µM various TPPP proteins were separately incubated with equal amount of various PrPs in 500 µl binding buffer (50 mM Tris-Cl, 100 mM NaCl, pH 8.0) at 4°C for 4 h, while same amount of GST protein was used as control. About 10 µl of Glutathione Sepharose 4B Agarose beads or Ni-NTA Agarose beads were added to the reaction solution and incubated at 37°C for 30 min with end-over-end mixing. After centrifugation at 3,000 rpm for 3 min, the supernatants were discarded and beads were washed with 500 µl washing buffer (50 mM Tris-Cl, 300 mM NaCl, pH 8.0) for three times. The complexes were separated on 15% SDS-polyacrylamide gel and electro-transferred to nitrocellulose filter membrane blocked with 5% nonfat milk in PBST (135 mM NaCl, 1.3 mM KCl, 3.2 mM Na_2_HPO_4_, 0.5 mM KH_2_PO_4_, 0.05% Tween 20, pH 7.4). PrP reactive band was detected with anti-PrP mAb 3F4 as the primary antibody and HRP-conjugated anti-mouse IgG as the second one, using ECL method (Perkin-Elmer, USA). TPPP reactive band was detected with anti-TPPP pAb as the primary antibody and HRP-conjugated anti-rabbit IgG as the second one, using ECL method.

### Immunoprecipitation

5 µM various GST-TPPP proteins were separately incubated with equal amount of His-PrP in 500 µl binding buffer containing 20 mM Tris-Cl, 200 mM NaCl, 10 mM aprotinin, pH 8.0, for 4 h, while equal amount of TPPP(1–219) protein alone was employed as control in parallel. After incubated with anti-TPPP pAb, anti-TPPP mAb or anti-PrP pAb at 4°C for 2 h, 10 µl protein G Sepharose (Roche, Switzerland) equilibrated by binding buffer was applied into the reactions and incubated for further 2 h. The Sepharose beads were precipitated at 3,000 rpm for 3 min and washed with 500 µl washing buffer (50 mM Tris-Cl, 500 mM NaCl, pH 8.0) for three times. The bound complexes were separated by 15% SDS-PAGE and transferred to nitrocellulose membranes. The bound PrP was detected by nti-PrP mAb 3F4, anti-TPPP pAb or anti-TPPP mAb in Western blot.

### Sedimentation experiments

Samples containing 0.5 mg/ml recombinant PrP were incubated for 60 min together with various TPPP at a PrP to TPPP molar ratio equal to 1∶2, and subsequently centrifuged for 10 min at 14,000×g. The pellets were resuspended in a volume of deionized water equal to the volume of the supernatants and analyzed by 15% SDS-PAGE.

### Transmission electron microscopy

To form fibril *in vitro*, 0.5 mg/ml synthetical peptide PrP106–126 were dissolved in the polymerization buffer (80 mM PIPES, pH 6.9, 0.5 mM MgCl_2_, 1 mM GTP, 5% glycerol) and incubated at 37°C for 72 h. To see the possible influences of TPPP on the formation of PrP106–126 fibril, different recombinant GST-TPPP proteins were mixed with the synthetical PrP106–126 at the molar ratio equal to 1∶2. After incubation at 37°C for 72 h, 10 µl of aliquot from each reaction was applied to copper grids for 1 min. Following negative staining performed with 2% (w/v) phosphotungstinic acid for 1 min. Images were obtained at ×97,000 magnification using a Phillips CEM100 transmission electron microscope.

### Thioflavin T (ThT) assay for amyloid fibril formation

The assembly of PrP106–126 fibrils was determined by ThT assay. Briefly, PrP106–126 were incubated with various GST-TPPP and GST in the same protocol described above at 37°C for 72 h. An aliquot (10 µl) of each reaction was mixed with 180 µl 50 mM glycine-OH (pH 8.5) containing 10 µM ThT (1 mM stock solution in water, Sigma T3516). The value of fluorescence was read by a spectrophotometer (F-4500 FL Spectrophotometer) at 485 nm, using an excitation wavelength of 440 nm.

### Cell culture and transfection

Cell lines HeLa and SHSY5Y were cultured in Dulbecco's modified Eagle's medium (DMEM) supplemented with 10% fetal calf serum. The monolayer Cells were transiently transfected or co-transfected with various plasmids using FugeneTM regent (Roche, Switzerland) according to the manufacture's protocol.

### Microtubule in the cultured cells

After transfected for 48 h, cells receiving different plasmids were fixed in 4% paraformaldehyde for 30 min at room temperature and then permeabilized with 0.5% Triton X-100 in PBS for 30 min. After blocking with 5% fetal bovine serum, cells were incubated with anti-a-tubulin (1∶200) antibody overnight at 4°C. After washing with PBS, cells were stained with Alexa Fluor 488 conjugated anti-mouse IgG (1∶200, Invitrogen, USA) for 1 h at room temperature. In parallel, the cells treated with 10 µM colchicines for 48 h were used as positive control. Fluorescently stained cells were analyzed with confocal laser scanning microscope (Leica ST2, Germany).

### Cell viability assays

HeLa cells were plated in 96-well trays and maintained in DMEM, and then transiently transfected or co-transfected with various recombinant plasmids. 24 and 48 h after transfection, cell viability was determined by a commercially Cell Counting Kit (CCK-8, Dojindo, Japan). 10 µl of CCK-8 reagent were added to each well and incubated at 37°C for 3 h, until the media turned yellow. Absorbance was measured at 450 nm in a spectrophotometer. Each experiment was performed in triplicate and repeated at least three times.

### Preparation of brain homogenates

Four Chinese golden hamsters inoculated intracerebrally with hamster-adapted scrapie strain 263K were enrolled in this study. The detailed clinical data of those scrapie strains were described previously [36]. Four hamsters of 80-day old were collected for health control. Brain homogenates of the hamsters were prepared described previously [37], birefly, brains from the scrapie-infected and health hamsters were washed in TBS (10 mM Tris HCl, 133 mM NaCl, pH 7.4) for three times, and then 10% (W/V) brain homogenates were prepared in lyses buffer (100 mM NaCl, 10 mM EDTA, 0.5% Nonidet P-40, 0.5% sodium deoxycholate, 10 mM Tris, pH 7.5) containing a mixture of protease inhibitors. The tissue debris was removed with low speed centrifugation at 2,000 g for 10 min and the supernatants were collected for further experiments.

### Statistical analysis

Quantitative analysis of immunoblot images was carried out using computer-assisted software Image Total Tech (Pharmacia). The values of each target blot were evaluated. All data are presented as the mean ± SD. Statistical analyses were performed using *SPSS* 17.0 statistical package. The differences of immunoblot images values were assessed by *T* test. The differences of OD450 values of CCK-8 between tested groups and vector control were assess by one-way ANOVA. Probabilities of less than 0.05 were considered to be statistically significant.
